# Iron-rich food consumption and associated factors among children aged 6–23 months in Sierra Leone: multi-level logistic regression analysis

**DOI:** 10.1186/s12889-023-16737-x

**Published:** 2023-09-15

**Authors:** Birhan Ewunu Semagn, Zenebe Abebe Gebreegziabher, Wondwosen Abey Abebaw

**Affiliations:** 1 Department of Public Health, School of Public Health, Asrat Woldeyes Health Science Campus, Debre Berhan University, Debre Berhan, Ethiopia; 2 Department of Epidemiology and Biostatistics, School of Public Health, Asrat Woldeyes Health Science Campus, Debre Berhan University, Debre Berhan, Ethiopia; 3 Department of Public Health, School of Public Health, College of Health Sciences, Woldia University, Woldia, Ethiopia

**Keywords:** Iron-rich food, Complementary feeding, Micro-nutrient deficiency, Children, Africa, Sierra Leone

## Abstract

**Background:**

Iron deficiency is the most common micronutrient deficiency worldwide. Also, iron deficiency is a significant public health problem in low- and middle-income countries. Thus, this study aimed to assess iron-rich food consumption and associated factors among children aged 6–23 months in Sierra Leone.

**Method:**

This study is a cross-sectional study based on data from the Sierra Leone Demographic and Health Survey dataset with a total weighted sample of 2622 children aged 6–23 months. Data cleaning, coding, and labeling were done using STATA version 14 software. A multilevel logistic regression model was employed to identify associated factors.

**Result:**

Almost half (53.38%) of children aged between 6–23 months consumed iron-rich foods. The odds of iron rich food consumption were high among children in the age group of 12–17 months (AOR = 4.81, 95% CI: 3.67, 6.31) and 18–23 months (AOR = 9.3, 95% CI: 6.55, 13.2), and who fed minimum acceptable diet (AOR = 22.5, 95% CI: 11.65, 43.46). Moreover, a child from a mother who had work (AOR = 1.49, 95% CI: 1.08, 2.06), and with a mother who had more than four ANC visits during her pregnancy of the most recent live birth (AOR = 1.87; 95%CI: 1.36—2.55) had higher odds of iron-rich food consumption compared to their counterparts. On the other hand, children who were breastfeeding (AOR = 0.72, 95% CI: 0.53, 0.97), and mothers aged 15–19 (AOR = 0.48, 95% CI: 0.27, 0.85) decreased the odds of iron rich food consumption.

**Conclusion:**

Consumption of iron-rich food is low among children aged 6–23 months in Sierra Leone. Iron-rich food consumption among children was significantly associated with maternal occupation, child’s age, child’s breastfeeding status, taking drugs for intestinal parasites, minimum acceptable diet, frequency, and timing of ANC, and region. Thus, special emphasis should be given to those children aged between 6–11 months, currently breastfeeding, children who did not get the minimum acceptable diet, and children from women who did not have work.

**Supplementary Information:**

The online version contains supplementary material available at 10.1186/s12889-023-16737-x.

## Introduction

Iron deficiency is one of the most prevalent public health problems affecting children and adolescents worldwide,and affects approximately two billion people globally [1, 2]. It’s the most common micro-nutrient deficiency [3, 4].In low- and middle-income countries under nutrition including iron deficiency is a major public health problem contributing to 45% of deaths among children under 5 years of age [5]. The first two years of life are the most essential period for child nutrition after which sub-optimal growth is hard to reverse [6]. The physiologic requirement of iron in early childhood is higher than during adulthood because of the high iron demand in blood volume expansion, muscle and tissue mass growth, brain growth, neurons-development, and immune response [7]. Iron deficiency may occur due to increased iron requirement (like childhood, and pregnancy), decreased intestinal iron absorption, blood loss, inflammation, and low iron-rich food intake [8]. Moreover, breastfeeding alone is no longer sufficient to meet iron demand and iron-rich meals should be introduced to make up for the shortcoming [9].

Childhood iron deficiency may result in adverse problems in cognitive function and psychomotor development [10, 11]. It may also induce or aggravate deficiency of other essential nutrients, which may result in a negative impact on the developing brain and other organs in infants [10]. When the body’s iron stores are too low, the body’s ability to produce hemoglobin will be depleted, and this lead to iron deficiency anemia with the possibility of ending with life-threatening cardio-vascular and renal complications [12]. Furthermore, iron deficiency will affect economic development by creating cognitive impairment, decreased work productivity, and death from severe anemia [13].

West Africa is one of the Sub-Saharan regions with the greatest prevalence of childhood malnutrition, with children's iron requirements not yet being reached [14]. Around 68% of children aged 6–59 months are anemic in Sierra Leone [15, 16]. Even though iron deficiency is frequently associated with iron deficiency anemia, the deficiency will also occur without manifesting anemia, meaning the number of children affected will be considerably higher with a suggested figure of at least double that of iron deficiency anemia [7, 8]. It may also lead to less clinically notable reductions in energy level, mental clarity,and overall capacity [3].

While a prior study on this topic was undertaken across multiple Sub-Saharan African countries, Sierra Leone was not included in that study [17]. Our study seeks to give important baseline evidence to future public health intervention planners by assessing the proportion and correlates related to iron-rich food consumption at the country level. Children’s age, maternal education, religion, household wealth, and feeding customs are some of the variables that influence children’s consumption of iron-rich food [17–19].

Children’s diets in developing nations are frequently characterized by a lack of variety, inconsistence quality, and fewer nutrient-dense foods. Infants and children have poor diets, even though their iron needs are significant between the age of 6 and 11 months due to rapid growth [20].

Many of the micronutrient deficiencies are preventable through nutrition education and the consumption of a healthy diet containing diverse foods [3]. Because of the association between therapeutic iron intervention and increased susceptibility to malaria, respiratory and gastrointestinal infections, as well as reshaping of the intestinal microbiome, there is a need to increase iron intake from iron-rich foods rather than relying on therapeutic iron supplementation [7].

For the designing, planning, and implementation of effective and sustainable interventions to improve the iron needs of children aged 6–23 months, an understanding of the determinants of iron-rich food consumption within a given context is one of the necessary steps to be taken. Despite the above facts, to the level of our knowledge, no study assesses the magnitude of iron-rich food consumption and its predictor among children using nationally representative data in Sierra Leone. Thus, the current study aimed to assess iron-rich food consumption and associated factors among children aged 6–23 months in Sierra Leone.

## Methods

### Study design, data source, and setting

This study is a cross-sectional study using data from the most recent nationally representative Sierra Leone Demographic and Health Survey (SLDHS) 2019. The 2019 Sierra Leone Demographic and Health Survey (SLDHS) were implemented by Statistics Sierra Leone (Stats SL) to provide up-to-date estimates of basic demographic and health indicators. Data collection took place from 15 May to 31 August 2019. The data were obtained from the Demographic and Health Survey (DHS) website (https://dhsprogram.com/) after submitting a request justifying the aim of the study. The Kids Record (KR) file of the SLDHS data set contains information related to pregnancy, postnatal care, immunization, health and nutrition data. Sierra Leone is one of the low -income sub-Saharan countries in West Africa, administratively divided into provinces, each province is subdivided into districts, each district is further divided into chiefdoms/census wards, and each chiefdom/census ward is divided into sections [15].

### Population and sampling procedure

**Fig. 1:**
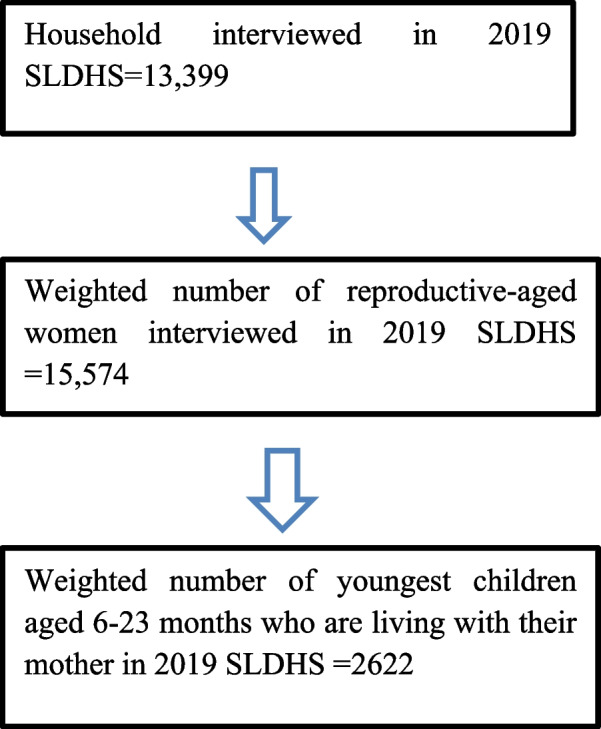
The sampling procedure, and the final sample size considered in this study to assess consumption of food rich in iron, and associated factors among children aged 6–23 months in Sierra Leone using the 2019 SLDHS dataset

All women aged 15–49 in the sample households were included in the 2019 SLDHS. The sampling procedure for the 2019 SLDHS was a two-stage stratified sampling. In the first stage, 578 Enumeration Areas (EAs) were selected with a probability proportional to EA size, and in the second stage’s selection, a fixed number of 24 households were selected in every cluster through equal probability systematic sampling, resulting in a total sample size of 13,399 interviewed households. Further information related to the population, study area, data collection, sampling procedures, and questionnaires used in the survey were detailed in the 2019 SLDHS Report [15].This study includes a weighted sample of 2622 youngest living children born in the 2 years preceding the survey who is living with the mother (KR file), and aged between 6–23 months Fig. 1.

### Study variables

#### Outcome variable

The outcome variable of the study was iron-rich food consumption among children of age 6–23 months. It was a binary outcome variable with the category of “Yes” if a child consumed iron-rich foods including meat (and organ meat), fish, poultry, and eggs in the last 24 h of the survey date, and “No” if a child consumed none of those iron-rich foods [17].

#### Explanatory variables

The potential predictors considered to have an association with iron-rich food consumption were chosen based on prior literatures [17–19]. Independent variables were grouped as Individual level variables,and Community level variables.

Individual level variables were household wealth index,household family size, sex of household head, maternal age,maternal-occupation,maternal education, media exposure, religion,mother internet use, husband/partner occupation,husband/paternal education, child age,sex of a child, birth order, breast feeding status, consumption of minimum acceptable diet,taking an intestinal drug, child medical illness(fever,Diarrhea,shortness of breath), distance to the health facility, frequency of Antenatal Care (ANC), Timing of ANC, place of delivery,and baby postnatal check within two days after birth.

Community-level variables included in this study were residence, region, community distance to the health facility, community ANC coverage, community maternal education level, community poverty level, and community media exposure. Only residence and region were captured primarily from SLDHS data, but all the other community-level variables were created by aggregating individual mothers’ characteristics within their clusters. The aggregates were computed using the proportions of women in each category of a given variable. To give a practical meaning we categorized the aggregated values of the cluster into two groups. Since all aggregates were not normally distributed, we categorized the aggregated values of the clusters in two groups based on the median value of the proportion for each respective variable. We used the same procedure for all aggregated community factors. (Supplementary 1).

### Data management and analysis

After downloading the data from the DHS website, data extraction, recoding, labeling, cross-tabulations, and analysis were done using STATA Version 14. In advance of conducting any statistical analysis, the data was weighted using sampling weight (v005), primary sampling units (v021), and strata (v022) to keep the representativeness of the survey and to get more reliable estimates.

Frequencies and percentages were used for descriptive statistics. Given the hierarchical structure of the demographic and health survey data, which violates the assumption of independent observation and equal variance [18, 21], we used multi-level logistic regression analysis for the inferential statistics. Interclass Correlation Coefficient (ICC) and Median Odds Ratio (MOR) were checked to assess whether there was clustering or not. Four separate models including the null model, model I (Individual-level variables), model II (Community-level variable), and Model III (models that include both individual and community level variables) were fitted. Because the model is nested, model comparisons were done using log likelihood ratio and deviance test. The best fitted model with the highest log -likelihood ratio and the lowest deviance was model III, which included both individual and community-level variables (Table 3).

Finally, both bivariable and multivariable multilevel logistic regression analysis was done using the best-fitted model. Variables with a *p*-value < 0.2 in the bivariable analysis were considered for multivariable analysis, and variables with a *p*-value < 0.05 in the multivariable analysis were considered as significant factors associated with iron-rich food consumption among children.

## Results

### Individual level background characteristics of respondents

This study includes a weighted number of 2622 children aged between 6–23 months in Sierra Leone. Table 1 shows the distribution of children’s iron-rich food consumption by background characteristics.

**Table 1:** Individual-level characteristics by iron-rich food consumption among children aged 6–23 months, SLDHS 2019, Sierra Leone (*n* ranges from 2156 to 2622)

Children given iron rich food				
**Variables**	**No**	**Yes**	**Total**	**Weighted**
	**%**	**%**	**%**	**Frequency**
**Household wealth-index**				
Poor	20.84	24.49	45.33	1,189
Middle	9.19	11.15	20.34	533
Rich	16.59	17.74	34.33	900
**Household family size**				
Greater than 5	27.91	33.58	61.49	1,612
Less than or equal to five	18.7	19.8	38.51	1,010
**Sex of household head**				
Male	35.42	41.12	76.54	2,007
Female	11.2	12.26	23.46	615
**Mother age in Year**				
15–19	4.93	4.99	9.92	260
20–34	31.15	37.1	68.25	1,790
35–49	10.54	11.29	21.83	572
**Occupation status**				
Not working	10.49	9.69	20.18	529
Working	36.13	43.69	79.82	2,093
**Education level**				
Low	32.04	36.77	68.81	1,804
High	14.58	16.62	31.19	818
**Exposure to either of the three media at least once a week**				
No	35.45	37.92	73.37	1,924
Yes	11.17	15.46	26.63	698
**Religion**^**a**^				
Christian	9.64	11.73	21.38	560
Islam	36.95	41.68	78.62	2,060
**Ever used the internet**				
No	42.73	48.91	91.64	2,403
Yes	3.89	4.47	8.36	219
**Husband occupation**^a^				
Not working	2.81	1.96	4.78	105
Working	42.95	52.28	95.22	2,100
**Husband attended secondary education and above**^**a**^				
No	28.72	34.78	63.5	1,369
Yes	16.75	19.76	36.5	787
**Child age in months**				
6–11	25.61	8.97	34.58	907
12–17	14.23	24.9	39.13	1,026
18–23	6.78	19.51	26.29	689
**Sex of child**				
Male	23.86	26.44	50.3	1,319
Female	22.76	26.94	49.7	1,303
**Birth order**				
1	11.32	11.99	23.3	611
2–4	23.96	26.98	50.94	1,336
> = 5	11.34	14.42	25.76	675
**Currently breastfeeding**				
No	6.93	17.93	24.87	652
Yes	39.68	35.45	75.13	1,970
**A Child with minimum acceptable diet**				
No	45.96	44.42	90.39	2,370
Yes	0.65	8.96	9.61	252
**A child took drug for intestinal parasite**^**a**^				
No	26.02	19.02	45.04	1,179
Yes	20.63	34.33	54.96	1,439
**A child had fever in last two weeks**				
No	37.75	41.03	78.78	2,066
Yes	8.86	12.36	21.22	556
**A child had diarrhea last two weeks**				
No	42.2	47.74	89.93	2,358
Yes	4.42	5.65	10.07	264
**A child had Short, rapid breaths**^**a**^				
No	43.98	50.19	94.17	2,466
Yes	2.6	3.23	5.83	153
**Distance to health facility**				
Big problem	22.27	27.52	49.79	1,306
Not a big problem	24.35	25.86	50.21	1,316
**Attended 4 + ANC visits**				
No	12.34	8.76	21.1	553
Yes	34.28	44.62	78.9	2,069
**Attended ANC in the first trimester of pregnancy**				
No	25.14	30.81	55.95	1,467
Yes	21.48	22.58	44.05	1,155
**Place of delivery**				
Non-health facility	5.88	8.27	14.16	371
Health facility	40.74	45.11	85.84	2,251
**New born postnatal check in the first 2 days**				
No	40.93	46.06	86.99	2,281
Yes	5.69	7.32	13.01	341

^a^Missing observations deleted from analysis

The majority of the children, 45.33%, 61.49%, and 76.54% respectively, were from households with poor wealth index, greater than five family members, and headed by males. Most of them (68.25%) were from mothers aged between 20–34 years old, mothers who had work (79.82%), with low educational level (68.81%), and with low media exposure (73.37%). Even though the majority (75.13%) of the children were breastfeeding at the time of data collection, and most of them (90.39%) didn’t consume the minimum acceptable diet. Furthermore, most of the mothers (78.9%) had four or more ANC visits during their pregnancy, although only (44%) of them started their ANC in their first trimester of pregnancy. Moreover, most (85.84%) of the children were delivered at health institutions but only 13% of the children had a new born post-natal check in the first two days of life (Table 1).

### Community level background characteristics of respondents

The majority of the respondents (65.55%) were rural residents, and from the eastern region (21.87%). About 53.59% of them were from a community in which distance to the health facility is not a problem for getting medical help, with good community ANC Coverage (81.36%), with low community poverty level (52.46), and low community media exposure (50.29) (Table 2).

**Table 2:** Community-level characteristics by iron-rich food consumption among children aged 6–23 months, SLDHS 2019, Sierra Leone (*N* = 2622)

Children given iron rich-food				
**Variables**	**No**	**Yes**	**Total**	**Weighted**
	**%**	**%**	**%**	**frequency**
**Type of place of residence**				
Urban	17.05	17.4	34.45	903
Rural	29.57	35.98	65.55	1,719
**Region**				
Eastern	13.28	8.59	21.87	573
Northern	8.64	11.09	19.73	517
Northwestern	6.53	12.21	18.74	491
Southern	9.33	11.93	21.26	557
Western	8.84	9.57	18.41	483
**Community distance to a health facility**				
A big problem	25.16	28.42	53.59	1,405
Not a big problem	21.45	24.96	46.41	1,217
**Community ANC coverage**				
lower community ANC	9.8	8.84	18.64	489
higher community ANC	36.82	44.54	81.36	2,133
**Community maternal education level**				
lower community maternal education	22.4	25.79	48.19	1,264
higher community maternal education	24.21	27.59	51.81	1,358
**Community poverty level**				
lower community poverty	24.54	27.92	52.46	1,376
higher community poverty	22.08	25.46	47.54	1,246
**Community media exposure**				
lower community media exposure	24.66	25.63	50.29	1,319
higher community media exposure	21.96	27.75	49.71	1,303

### Iron-rich food consumption

Overall, only half (53.38%) 95% CI (51%-55%) of children aged between 6–23 months consumed foods rich in iron.

### Random effect (community-level clustering) and model comparison

The random effect model has been assessed to identify the presence of community-level clustering. The result of the random effect model implies the presence of significant clustering given that the ICC value in the null model was 0.14, which indicates that about 14% of the total variation in iron-rich food consumption was attributable to the difference between clusters, moreover the value of MOR (95% CI) was 2 (1.72–2.27) that points to iron-rich food consumption among children significantly different between clusters. This means if we randomly select children aged between 6–23 from different clusters, children in the cluster with higher iron -rich food consumption had 2 times higher odds of consuming iron-rich food as compared with those children in clusters with lower iron-rich food consumption. Regarding model fitness, the final model (Model III) with the lowest deviance/highest log likelihood was the best-fitted model (Table 3).

**Table 3:** Random effect model and model fitness comparison for factors associated with iron-rich food consumption among children of age 6–23 months in Sierra Leone (Weighted *n* = 2622)

Parameter	Null Model	Model I	Model II	Model III ^a^
ICC	0.14	0.15	0.1	0.1
MOR (95%CI)	2(1.72–2.27)	2.1(1.7–2.5)	1.8(1.5–2.1)	1.9(1.5–2.3)
Model Comparison				
Log likelihood	-1797.15	-1201.84	-1768.71	-1181.18
Deviance	3594.3	2403.68	3537.42	2362.36

^a^The best-fitted model with high Log-likelihood, and low deviance

### Individual and community-level factors associated with iron-rich food consumption among children of age 6–23

The variables that passed the screening (*p*-value < 0.2) in the bivariable analysis were household family size, mother’s age in years, occupational status of the mother, media exposure, husband occupation, child’s age in months, birth order, breastfeeding status, use of a drug for intestinal parasites, a child with minimum acceptable diet, had a fever recently, had diarrhea recently, frequency of ANC visit, the timing of ANC, residence, region, community distance to the health facility, community ANC coverage, and community media exposure. Finally, nine variables made up the final adjusted multivariable multilevel regression model as determinants of iron-rich food consumption (*p*-value < 0.05) (Table 4).

**Table 4:** Bivariable, and multivariable multilevel logistic regression analysis of individual and community level factors associated with iron-rich food consumption among children of age 6–23 months in Sierra Leone (Final sample size after pairwise deletion = 2222)

**Variables**		**COR**	**CI**	**AOR**	**95%CI**
**Household wealth-index**	Poor	1			
	Middle	0.96	0.76—1.22		
	Rich	0.92	0.72—1.16		
**Household family size**	> 5	1		1	
	< = 5	0.85***	0.71—1.02	0.84	0.67—1.05
**Sex of household head**	Male	1			
	Female	0.88	0.72—1.09		
**Mother's age in Year**	15–19	1		1	
	20–34	1.28***	0.95—1.72	0.61	0.37—1.01
	35–49	1.11	0.80—1.55	0.48*	0.27—0.85
**Occupation status of Mother**	Not working	1		1	
	Working	1.36***	1.09—1.71	1.49*	1.08—2.06
**Mother Education secondary and above**	No	1			
	Yes	1.03	0.85—1.26		
**Exposure to either of the three media at least once a week**	No	1		1	
	Yes	1.22***	0.99—1.51	1.08	0.81—1.45
**Religion**	Christian	1			
	Islam	0.92	0.74—1.16		
**Ever used the internet**	No	1			
	Yes	1.22	0.87—1.71		
**Husband occupation**	Not working	1		1	
	Working	1.72***	1.09—2.71	1.35	0.78—2.34
**Husband attended secondary education and above**	No	1			
	Yes	0.97	0.79—1.20		
**Child age in months**	6–11	1		1	
	12–17	7.05***	5.53—8.98	4.81***	3.67—6.31
	18–23	13.19***	9.93—17.50	9.30***	6.55—13.20
**Sex of a child**	Male	1			
	Female	1.09	0.91—1.29		
**Birth order**	1	1		1	
	2–4	1.05	0.85—1.31	1.11	0.79—1.56
	> = 5	1.19***	0.93—1.53	1.31	0.87—1.98
**Currently breastfeeding**	No	1		1	
	Yes	0.27***	0.22—0.34	0.72*	0.53—0.97
**Drug for intestinal parasite in the last 6 months**	No	1		1	
	Yes	2.82***	2.33—3.41	1.40**	1.12—1.75
**Child with minimum acceptable diet**	No	1		1	
	Yes	17.39***	10.19 -29.67	22.50***	11.65—43.46
**Had fever in last two weeks**	No	1		1	
	Yes	1.17***	0.94—1.45	1.13	0.86—1.48
**Had diarrhea last two weeks**	No	1		1	
	Yes	1.13	0.85—1.52	1.27	0.87—1.85
**Short, rapid breath**	No	1			
	Yes	0.89	0.61—1.30		
**Distance to health facility**	A big problem	1		1	
	Not a big problem	0.85***	0.70—1.03	0.84	0.66—1.06
**Attended 4 + ANC visits**	No	1		1	
	Yes	2.04***	1.62—2.55	1.87***	1.36—2.55
**Attended ANC < 4 months of pregnancy**	No	1		1	
	Yes	0.87***	0.72—1.04	0.80*	0.64—0.99
**Place of delivery**	Non-health facility	1			
	Health facility	0.91	0.70—1.20		
**New born postnatal check in the first 2 days**	No	1			
	Yes	1.06	0.81—1.38		
**Type of place of residence**	Urban	1			
	Rural	1.20***	0.94—1.52	1.13	0.81—1.60
**Region**	Eastern	1			
	Northern	2.26***	1.61—3.17	2.37***	1.64—3.42
	Northwestern	3.17***	2.22—4.51	3.01***	2.02—4.48
	Southern	2.09***	1.50—2.90	1.95***	1.35—2.82
	Western	1.79***	1.26—2.55	1.84*	1.10—3.07
**Community distance to a health facility**	A big problem	1			
	Not a big problem	1.05	0.84—1.33		
**Community ANC coverage**	Low	1			
	High	1.39***	1.05—1.86	1.02	0.70—1.48
**Community maternal education level**	Low	1			
	High	1.02	0.81—1.28		
**Community poverty level**	Low	1			
	High	1.02	0.81—1.28		
**Community media exposure**	Low	1			
	High	1.24***	0.98—1.55	1.28	0.96—1.70

COR *** *p* < 0.2

AOR *** *p* < 0.001

^**^ *p* < 0.01

^*^ *p* < 0.05

In the multivariable multilevel logistic regression analysis, maternal age, maternal occupation, children’s age, children’s breastfeeding status, children who took drugs for intestinal parasites, minimum acceptable diet, frequency, and timing of ANC, and region were found to be significant factors associated with iron-rich food consumption.

A child from women in the age group of 35–49 had a 52% decreased odds of iron-rich food consumption compared to a child from women in the age group 15–19 (AOR = 0.48, 95% CI: 0.27, 0.85). The odds of iron-rich food consumption were increased by 49% among children from mothers who had work compared to children from women who did not have work (AOR = 1.49, 95% CI: 1.08, 2.06).

The odds of iron-rich food consumption among the child in age group of 12–17 months, and 18–23 months were 4.81 (AOR = 4.81, 95% CI: 3.67, 6.31), and 9.3 (AOR = 9.3, 95% CI: 6.55, 13.2) times higher as compared with a child in the age group of 6–11 months.

Another result from this study is children who were breastfeeding during the period of data collection had 28% lower odds of iron-rich food consumption compared to children who were not breastfeeding during the period of data collection (AOR = 0.72, 95% CI: 0.53, 0.97).

The study also shows the odds of iron-rich food consumption among children who took drugs for intestinal parasites were increased by 40% (AOR = 1.40, 95% CI: 1.12, 1.75) compared to children who did not take drugs for intestinal parasites.

Moreover, the odds of iron-rich food consumption among children who took the minimum acceptable diet were 22.5 times higher than children who did not take the minimum acceptable diet (AOR = 22.5, 95% CI: 11.65, 43.46).

Looking at the frequency of ANC visits the mother made in the most recent pregnancy, children who were from women who had more than four ANC visits had 1.87 times (AOR = 1.87; 95%CI: 1.36—2.55) higher odds of consuming iron-rich food compared to their counterparts.

Children from mothers who had ANC visits in the first trimester of pregnancy at the most recent live birth were 20% times less likely to consume iron-rich food than a child from mothers who did not have an ANC visit in the first trimester of their pregnancy (AOR = 0.80;95%CI = 0.64 – 0.99).

Regarding community-level covariates, we found higher odds of iron-rich food consumption among children who resided in Northern (AOR = 2.37; 95%CI: 1.64 – 3.42), Northwestern (AOR = 3.01; 95%CI: 2.02 – 4.48), southern (AOR = 1.95; 95%CI: 1.35 – 2.82), and western (AOR = 1.84; 95%CI: 1.10 – 3.07) regions as compared with children who reside in Eastern region Table 4.

## Discussion

Sierra Leone is one of the West African Countries with the highest malnutrition rates in the world [22]. This study aimed to assess iron-rich food consumption and associated factors among children of age 6–23 months in Sierra Leone using data from the most recent SLDHS 2019. According to this study, 53% of children consumed iron-rich food within 24 h of the survey date. This is higher than studies conducted in Ethiopia [18, 23], a study conducted in other Sub-Saharan African Countries (42.1%),and in Rwanda (23.56%) [24].This discrepancy might be due to the difference in culture, and practice of child feeding between mothers [17]. However, it is lower than from the study conducted in Australia (82%0.6) [25], Ireland (90%) [24], and the Pacific (62.5%) [26]. Such lower consumption of iron-rich foods in Sierra Leone could be attributed to household food insecurity and poor economic status which makes iron-rich foods unaffordable [26, 27].

In multivariable multilevel logistic regression analysis maternal occupation, child’s age, child’s breastfeeding status, taking drugs for an intestinal parasite, minimum acceptable diet, frequency, and timing of ANC, and region were found to be statistically significant factors associated with iron-rich food consumption among children of age 6–23 month in Sierra Leone.

In line with a study conducted in sub-Saharan regions out of Sierra Leone [17], maternal occupation is found to have a significant association with iron-rich food consumption, which means children from women who have work had a higher probability of consuming iron-rich food as compared with their counterparts. This might be due to the chance of having a good income as a result of having work so that mothers will feed their children with iron-rich food [28].

Children’s age is a significant predictor of iron-rich food consumption. Children ages 12–17 and 18–23 months were more likely to consume iron-rich food as compared with children ages 6–11 months. Consistence with this, different studies report a high prevalence of iron deficiency among children of younger age [29, 30]. This is in line with a study conducted in Ethiopia, and other Sub-Saharan African regions [17, 18]. A possible justification for this might be late introduction of complimentary feeding.

Children’s breastfeeding status during the time of the survey was also a significant factor associated with iron-rich food consumption, children who fed breast milk within 24 h of the survey date were less likely to consume iron-rich food.The possible justification for this discrepancy might be because of not introducing complimentary food at an appropriate age [31].

A Child who took drugs for intestinal parasites had a higher chance of consuming iron-rich food. This is in line with a study conducted in sub-Saharan regions [17, 31]. This might be related to the motivation and commitment of a woman in providing iron-rich food for her child [17].

A minimum acceptable diet predicts iron-rich food consumption. This is consistence with a study conducted in Ethiopia that revealed as children from households with increased dietary diversity consumed more iron-rich food as compared with their counterparts [23]. This might be because children with a minimum acceptable diet are more likely to get diversified foods including iron-rich foods like meat, fish, and eggs.

ANC service utilization frequency and timing of ANC is associated with iron-rich food consumption. This goes with a study conducted in other Sub-Saharan African Countries that revealed more frequent ANC increased the likely of iron-rich food consumption among children [17]. Children of a woman who had more than 4 ANC visits were more likely to have iron-rich food as compared with their counterparts; this might be due to the possibility of frequent contact with health professionals so that a woman might have sufficient nutritional counseling.

Moreover, children from the north, northwest, west, and south regions were more likely associated with iron-rich food consumption compared with children from the eastern region. This is consistence with a study conducted in Ethiopia, that revealed variation in children iron-rich food consumption across regions [18]. This might be due to the difference in cultural, and child-feeding practices, as well as the availability of iron rich foods between regions.

This study has the strength of addressing an unaddressed topic in Sierra Leone using nationally representative data, as well as we use an advanced model to estimate both individual and community-level variables. Due to the secondary nature of the data, we are unable to incorporate if a child consumes iron-rich food from plant sources, and if a child consumes foods that enhance or decrease iron absorption.

## Conclusion

The study revealed that only half of the children aged 6–23 months consumed iron-rich food in Sierra Leone. Iron-rich food consumption among children was significantly associated with maternal occupation, child’s age, child’s breastfeeding status, taking drugs for intestinal parasites, minimum acceptable diet, frequency and timing of ANC, and region. Thus, special emphasis should be given to those children from women who did not have work, aged between 6–11 months, currently breastfeeding, and children who did not get the minimum acceptable diet. Furthermore, implementing public health programs that target to enable women to have more frequent ANC follow-ups may be an effective way to increase iron-rich food consumption. We recommend future researchers access the geographic distribution of poor iron-rich food consumption in the study area.

### Supplementary Information

**Additional file 1.**

## Data Availability

The datasets analyzed during the current study are available at the DHS website https://dhsprogram.com/data/dataset_admin/index.cfm
