# Supplementary material for: Iron-rich food consumption and associated factors among children aged 6–23 months in Sierra Leone: multi-level logistic regression analysis
Source: BMC Public Health. 2023 Sep 15;23:1793. doi: 10.1186/s12889-023-16737-x (PMC10503148; doi:10.1186/s12889-023-16737-x)
Supplement: Supplementary file 1 — Additional file 1. [file 12889_2023_16737_MOESM1_ESM.docx]

| **Individual level variables** |
| --- |
| *Household Wealth Index*: The datasets contained a wealth index that was created using principal components analysis coded as “poorest”, “poorer”, “Middle”, “Richer”, and “Richest in the SLDHS data set.” For this study, we recoded it into three categories “poor” (includes the poorest and the poorer categories), “middle”, and “rich” (includes the richer and the richest categories) |
| *Household family size*: The family size of the women's household re-coded into two categories with values of“0” for a family size greater than 5, and “1” for a family size of less than or equal to 5. |
| *Sex of household head*: the variable sex of household head was recorded as male and female in the dataset and we used without modifying it. |
| *Maternal age*: the age of the women was re-coded into three categories with values of “0” for 15–24, "1" for 25-34 ,and “1” for 35-49 |
| *Maternal Occupation*: occupational status of women were recoded as Not working "0" if a women did not engage in any work, or Working "1" if a women had something to work |
| *Maternal Education*: this is the educational level a woman achieved re coded as “0” low if mother's educational level was primary and below, and "1" high for women with secondary and higher education |
| *Media Exposure*: was recoded as yes "1" if a women had an exposure to either of the three media(radio, television, newspaper) at least once a week and no "0" if not |
| *Religion:* The variable religion was recorded as Christian "1", and Islam "0”. |
| *Mother Internet use*: this variable was recoded as No "0" if a women never used internet, or yes "1" otherwise |
| *Husband/partner Occupation*: occupational status of respondent's husband were recoded as Not working "0" if he did not engage in any work, or Working "1" if a he had something to work |
| *Husband/paternal Education:* this is the educational level a husband achieved re coded as “0” low if his educational level was primary and below, and "1" high for husband with secondary and higher education |
| *Child age*: the age of the child was re-coded into three categories with values of “0” for 6-11 months, "1" for 12-17 , and “2” for 18-23 months. |
| *Sex of a child*: This was recorded as male and female in the dataset and we used without modifying it. |
| *Birth order:* was re-coded into three categories with values of “0” for first order, "1" for 2-4, and “2” for >=5. |
| *Current breast feeding status*: Breastfeeding status refers to a “24-hour” period (yesterday and last night) preceding the survey was recoded as yes "1"if a child got breast feed, and no "0" if not |
| *Consumption of Minimum acceptable diet*: a composite variable created by combining minimum dietary diversity and minimum meal frequency children fed during the day or night preceding the survey and coded as yes "1",and no "0" |
| *Taking intestinal drug:* this was recorded as 1 "yes" if a child took an intestinal drug with in six month preceding the survey, and 0 "no" if not |
| *Fever, Diarrhea, and shortness of breath*: each of these medical illness was recorded as 1 "yes" if a child experienced them within two weeks preceding the survey, and 0 "no" if not |
| *Distance to the health facility:* This variable was recorded as "1” big problem and "0" not a big problem in the dataset and we used without modifying it. |
| *Frequency of Ante Natal Care (ANC):*The number of ANC visits during pregnancy were categorized into two groups and recoded as 1 "yes “if a woman have greater than or equal to four ANC, and 0 "No “if a woman didn't have greater than or equal to four ANC visit for the most recent live birth |
| *Timing of ANC*: The timing of ANC visits were categorized into two groups and recoded as 1 "yes “if a woman had ANC visit in the first trimester of her pregnancy to the most recent live birth , 0 "No “if a woman didn't have ANC visit in the first trimester of their pregnancy. |
| *Place of Delivery*: dichotomized as a health facility "1" (if a woman gives birth in public, private, or NGO health institutions) and a Non-health facility "0" (if a woman gives birth either in home or any other places) |
| *Baby post natal check within the first two day of life*: this was recoded as yes "1"and no "0" |
| **Community Level Variables** |
| *Residence*: coded as Urban "1",and "0" rural |
| *Region*: The variable region was recorded as eastern, northern, northwestern, southern, and western in the dataset and we used it without change |
| *Community distance to health facility:* This variable was derived from the individual values for distance to health facility. The aggregate values were grouped into two categories based on the median value of the proportion of women report distance to health facility as a big problem in each clusters “1” distance a big problem, “0”distance not a big problem. |
| *Community ANC coverage*: This variable was derived from the individual values for ANC utilization. The aggregate values were grouped into two categories based on the median value of the proportion of ANC utilization in each clusters “1” higher ANC utilizing “0” lower ANC utilizing. |
| *Community maternal education level:* This variable was derived from the individual values for women educational level. The aggregate values were grouped into two categories based on the median value of the proportion of women report secondary and higher education in each clusters “1”higher educational attainment, “0”lower educational attainment. |
| *Community poverty level:* This variable was derived from the individual values for women’s household wealth index. The aggregate values were grouped into two categories based on the median value of the proportion women from the two lowest wealth quintiles in a given community. The two values for the community poverty level were “1” higher poverty “0”lower poverty |
| *Community media exposure:* This variable was derived from the individual composite variable media exposure. The aggregate values were grouped into two categories based on the median value of the proportion women exposed to media in a given community. The two values for the community media exposure level were “1” higher media exposure, and “0”lower media exposure. |
